# Postural health education in schools: teachers' perspectives on well-being, inequities, and institutional constraints

**DOI:** 10.1080/17482631.2026.2674331

**Published:** 2026-05-14

**Authors:** Marco A. García-Luna, António Camilo Cunha, Carmen Manchado, Juan M. Cortell-Tormo

**Affiliations:** a Department of General and Specific Didactics, Faculty of Education, University of Alicante, Alicante, Spain; b Instituto de Educação e Psicologia, Universidade do Minho, Braga, Portugal

**Keywords:** Postural health education, physical education teachers, health literacy, school health promotion, qualitative research, educational inequities

## Abstract

**Purpose:**

Postural health remains an underexamined dimension of school-based health education, despite its relevance for students’ wellbeing and long-term musculoskeletal health. This study explores how Physical Education teachers conceptualise and implement postural health education, and which factors shape its enactment.

**Methods:**

A qualitative descriptive–interpretive design was adopted. Semi-structured interviews were conducted with 30 Physical Education teachers from primary and secondary schools in Spain. Data were audio-recorded, transcribed verbatim, and analysed using reflexive thematic analysis within a constructivist framework.

**Results:**

Five interrelated themes captured a tension between teachers’ strong valuation of postural health and its marginal curricular status. These themes reflected (i) its recognised but limited curricular integration; (ii) fragmented conceptualisations and uneven pedagogical content knowledge; (iii) predominantly sporadic and unstructured implementation; (iv) insufficiently practice-oriented training; and (v) a strong demand for practical and institutional support. Conceptualisations ranged from hygiene-oriented approaches to more integrative understandings linked to motor control and physical literacy. Implementation was shaped by limited training, scarce resources, and low institutional prioritisation.

**Conclusions:**

Findings reveal a gap between the perceived importance of postural health and the structures supporting its implementation. Addressing this gap requires context-sensitive approaches that strengthen pedagogical support and curricular alignment.

## Introduction

Musculoskeletal disorders and spinal pain are an increasing public health concern among school-aged children, with up to 30% reporting recurrent back or neck discomfort by mid-adolescence (Araújo-Lima et al., 2023; Salsali et al., 2023). These symptoms are associated with prolonged sedentary behaviour, inadequate school furniture, and postural habits acquired early in life, factors that undermine physical health and are linked to reduced academic engagement and quality of life (Araújo-Lima et al., 2023; Valenciano et al., 2020). Beyond physical discomfort, postural problems can also influence students’ emotional wellbeing, participation, and perceived quality of life, positioning postural health as a relevant yet often overlooked dimension of school-based wellbeing promotion (Kamper et al., 2016).

Despite its relevance, postural health is rarely framed as a core component of health literacy or systematically integrated into school health education (Kickbusch, 2001; Nutbeam, 2000; Nutbeam, 2025). Its limited visibility within curricular frameworks reflects broader challenges in how health-related knowledge is prioritised, legitimised, and enacted within educational systems. In this sense, curricular decisions do not merely organise content but actively shape students’ opportunities to access health-promoting knowledge, with potential implications for equity in health education.

Poor posture in school-aged children is associated with a range of health risks, including chronic musculoskeletal pain, reduced spinal mobility, impaired respiratory function, and long-term postural deformities (Araújo-Lima et al., 2023; Kamper et al., 2016; Valenciano et al., 2020). Beyond these physical risks, postural health education has been linked to broader developmental outcomes: adequate postural habits and body awareness are associated with improved proprioception, enhanced physical self-efficacy, and better motor control (Araújo-Lima et al., 2023; Valenciano et al., 2020). Moreover, evidence suggests that postural competence may influence cognitive engagement, classroom attention, and social interaction, particularly when poor posture is associated with fatigue or discomfort (Kamper et al., 2016). These multidimensional benefits underscore the case for treating postural health not merely as a hygiene concern but as an integral component of health literacy and physical literacy in school-based education (Kickbusch, 2001; Nutbeam, 2000; Nutbeam, 2025; Whitehead, 2010).

A growing body of research has examined the effectiveness of school-based postural education programmes, which typically combine ergonomic instruction, strengthening exercises, and proprioceptive training. Systematic reviews suggest that well-designed interventions can produce improvements in biomechanical outcomes and self-reported behaviours (Anyachukwu et al., 2024; Araújo-Lima et al., 2023; Brink et al., 2022; Dugan, 2018; Fisher & Louw, 2022; Kovacs et al., 2022). For instance, ergonomic education combined with exercise has been shown to reduce the prevalence of back pain and improve postural awareness in primary and secondary school students (Anyachukwu et al., 2024; Araújo-Lima et al., 2023; Brink et al., 2022). Proprioceptive training and movement-based interventions have also demonstrated improvements in spinal alignment and body mechanics during seated and standing tasks (Dugan, 2018; Fisher & Louw, 2022; Kovacs et al., 2022). However, this body of literature has primarily focused on programme design, implementation under controlled conditions, and short-term outcomes. Far less attention has been paid to how postural health education is interpreted, prioritised, and enacted within everyday school practice, particularly from the perspective of teachers who are responsible for its delivery.

Existing studies have explored teachers’ ergonomic knowledge and training experiences (Chacón-Borrego et al., 2018; Vidal-Conti et al., 2021), but these approaches provide limited insight into the complex interplay between teachers’ beliefs, pedagogical knowledge, and the institutional conditions that shape their practices. Moreover, increases in theoretical knowledge following professional development do not necessarily translate into sustained pedagogical change, especially in the absence of ongoing support, practical resources, and alignment with curricular expectations (Ávalos-Bevan, 2011; Desimone, 2009; Vidal-Conti et al., 2021). As a result, important questions remain about how postural health education is integrated into real-world educational contexts and why its implementation remains inconsistent.

Addressing these gaps requires moving beyond an intervention-focused perspective and examining postural health education as a socially and institutionally mediated practice. From this perspective, teachers are not merely implementers of predefined programmes but active agents who interpret, adapt, and prioritise content within specific pedagogical and organisational contexts. Concepts such as pedagogical content knowledge (PCK) (Ball et al., 2008; Shulman, 1987), physical literacy (Whitehead, 2010), and health literacy (Kickbusch, 2001; Nutbeam, 2000, 2025) provide useful lenses to explore how teachers understand and translate postural health into meaningful learning experiences. These constructs are not treated as separate variables but as interrelated analytical lenses that jointly inform the interpretation of teachers’ perspectives within institutional contexts. Specifically, PCK provides a framework for understanding how teachers transform subject-matter knowledge into accessible and pedagogically meaningful learning experiences (Ball et al., 2008; Shulman, 1987); physical literacy situates postural education within a broader conception of embodied, lifelong movement competence that encompasses motivation, confidence, and physical knowledge (Whitehead, 2010); and health literacy highlights the role of educational processes in enabling individuals to access, appraise, and act upon health-related information within social and institutional settings (Kickbusch, 2001; Nutbeam, 2000; Nutbeam, 2025). Together, these lenses allow for a multi-dimensional interpretation of both the knowledge demands and the structural conditions shaping how postural health education is conceptualised and enacted in schools. At the same time, institutional structures—including curricular frameworks, school cultures, and resource allocation—shape what forms of health knowledge are legitimised, taught, and assessed within school systems.

Qualitative inquiry is particularly well suited to examining these dynamics, as it enables an in-depth exploration of teachers’ meaning-making processes, professional reasoning, and situated practices (Braun & Clarke, 2006, 2019). Within this constructivist orientation, reflexive thematic analysis (RTA) allows for an interpretative engagement with participants’ accounts, recognising knowledge as co-constructed between participants and researchers. By foregrounding teachers’ perspectives, qualitative research can illuminate how health education is enacted in everyday contexts and how structural and cultural factors influence both teaching practices and students’ access to health-related knowledge.

Accordingly, this study draws on semi-structured interviews with Physical Education (PE) teachers to explore how postural health education is conceptualised and implemented within school settings. Specifically, the study addresses the following research questions:

(i) How do PE teachers conceptualise postural health education within school contexts?
(ii) How is postural health education enacted and integrated into PE curricula and practices?
(iii) What pedagogical, institutional, and contextual factors shape teachers’ implementation of postural health education?

PE teachers were selected as the focus of this study given their central role in health-related instruction within the formal school curriculum, and their unique position to integrate postural health content across both primary and secondary educational stages. By situating teachers’ perspectives within broader institutional and pedagogical contexts, this study contributes to global discussions on the social and structural determinants of health education, highlighting how educational systems may shape—and potentially reproduce—inequities in access to health-related knowledge (Kickbusch, 2001).

## Methods

### Study design

This study adopted a qualitative descriptive–interpretive design to explore how PE teachers conceptualise and enact postural health education within school settings. A qualitative approach was considered appropriate given the study’s aim to examine teachers’ meanings, experiences, and situated practices in relation to health education. The study was grounded in a constructivist epistemology, which assumes that knowledge is socially produced and shaped by individuals’ experiences, professional contexts, and institutional environments (Guba & Lincoln, 1994). From this perspective, teachers’ accounts are understood not as objective representations of reality, but as situated interpretations that reflect their pedagogical reasoning and the conditions in which they work.

RTA, as developed by Braun and Clarke (2006, 2019), was selected as the analytical approach because of its flexibility and its suitability for identifying and interpreting patterns of meaning across participants’ narratives. RTA aligns with a constructivist orientation by emphasising the active role of the researcher in knowledge production and by prioritising depth of interpretation over quantification or frequency-based analysis. Within this constructivist orientation, RTA allows for an interpretative engagement with participants’ accounts, recognising knowledge as co-constructed between participants and researchers. This approach enabled an in-depth exploration of how teachers make sense of postural health education, how they translate it into practice, and how these processes are shaped by broader pedagogical and institutional contexts.

### Participants and sampling

A purposive sampling strategy was employed to recruit PE teachers with relevant experience in health-related teaching. Participants were selected based on the following criteria: (i) being a qualified PE teacher; (ii) having at least three years of teaching experience related to health or postural education; and (iii) currently teaching in primary or secondary education during the 2024–2025 academic year. A total of 30 teachers (15 from primary and 15 from secondary education; 19 men, 11 women; age range 29–58 years, M = 41.4) participated in the study, with teaching experience ranging from 5 to 32 years. Although detailed school-level indicators (e.g., socio-economic composition or school size) were not systematically collected, participants were recruited from a range of public and semi-public schools across the Valencian Community, Spain (Alicante, Valencia and Castellón). This allowed for variation in institutional contexts and teaching conditions, which was considered sufficient for the exploratory and interpretative aims of the study.

Recruitment was conducted through direct email contact with schools and through existing professional and educational networks. School administrations were first contacted to facilitate access, and teachers who met the inclusion criteria were invited to participate voluntarily. Efforts were made to ensure variation in teaching experience, educational stage, and school context in order to capture a range of perspectives. Sampling and data collection were conducted iteratively, allowing emerging insights to inform subsequent recruitment. Sample adequacy was guided by the principle of information power, whereby the relevance of participants, the specificity of the research aim, and the richness of the data informed decisions about sample size.

In addition, and in response to conventional expectations in qualitative research, data saturation was considered as a process of analytical sufficiency rather than a fixed numerical threshold. Saturation was understood to be reached when additional interviews did not generate substantially new interpretative insights and when the developing thematic structure was sufficiently rich and coherent to address the research questions. In practice, this point was observed in the later stages of data collection, when subsequent interviews consistently reinforced previously identified patterns rather than contributing novel interpretative dimensions. This judgement was made through ongoing comparison between new data and the evolving thematic framework during the analysis, ensuring that the final themes were sufficiently developed in depth, coherence, and variation to address the research questions.

### Data collection

Data were collected through semi-structured interviews conducted online via Google Meet between October 2024 and March 2025. This format enabled access to participants across different geographical locations while maintaining flexibility and depth in the data collection process. An interview guide was developed to explore five domains aligned with the study aims and grounded in prior literature on postural education, teacher professional development, and school health integration (Araújo-Lima et al., 2023; Ávalos-Bevan, 2011; Chacón-Borrego et al., 2018; Desimone, 2009; Vidal-Conti et al., 2021): (i) perceived importance and curricular relevance of postural education; (ii) conceptual understanding and self-assessed knowledge; (iii) teaching practices and perceived barriers; (iv) initial and continuing training; and (v) perceived needs and institutional support. The guide consisted of 10 open-ended questions (two per domain). Two experts in PE pedagogy reviewed the guide, and it was piloted with two non-participant teachers to ensure clarity and relevance. The original Spanish questions were translated into English for reporting and are included in the supplementary materials alongside the coding framework. Spain's PE curriculum is regulated at both national and regional levels, with the Valencian Community operating under its own curricular framework. This context is relevant as it shapes the institutional conditions within which teachers work and may influence both their conceptualisations of postural health and the structural constraints they face. Conducting interviews in Spanish, the participants' native language, was considered essential to enable in-depth, nuanced communication and minimise linguistic barriers to self-expression.

Interviews lasted between 45 and 70 minutes, were audio-recorded with participants’ consent, and transcribed verbatim for analysis. They were conducted in Spanish by the lead researcher, whose background in PE, postural education, and biomechanics facilitated rapport with participants and an in-depth understanding of the topic. At the same time, this positionality required ongoing reflexive awareness of potential assumptions and their influence on data collection.

### Data analysis

Data were analysed using RTA following Braun and Clarke’s (2006, 2019) six-phase framework: (i) familiarisation with the data; (ii) generation of initial codes; (iii) construction of themes; (iv) review of themes; (v) definition and naming of themes; and (vi) production of the report. Analysis was primarily inductive, allowing patterns of meaning to emerge from the data while remaining informed by the study’s conceptual orientation. A detailed codebook with definitions and examples is provided in the supplementary materials. The analysis was conducted manually, without the use of qualitative data analysis software. This decision was consistent with the interpretative and constructivist orientation of the study, and is in keeping with the view that close, direct engagement with the data can enhance reflexive depth and analytical immersion (Braun & Clarke, 2006, 2019).

The analytical process began with repeated reading of the transcripts to achieve familiarisation. Initial codes were then generated to capture meaningful features of the data across the dataset. Coding was conducted independently by two researchers, followed by iterative discussions to refine codes and explore different interpretative possibilities. These discussions did not aim to achieve consensus or inter-rater reliability, but rather to enhance reflexivity and analytical depth.

Codes were subsequently organised into candidate themes, which were progressively reviewed, refined, and redefined through an iterative process. This involved examining relationships between codes, collapsing overlapping themes, and ensuring that each theme captured a coherent and meaningful pattern in relation to the research questions. The final analysis resulted in five themes that represent interpretative patterns across participants’ accounts. In line with a reflexive thematic approach, themes are understood as the product of an interpretative process rather than as categories derived from frequency counts. For this reason, the analysis prioritised the significance and explanatory power of themes over their prevalence. The assessment of data saturation was closely linked to this iterative analytical process, whereby ongoing coding and theme development allowed the research team to identify the point at which no substantially new interpretative insights were emerging.

### Trustworthiness

The rigour of the study was ensured through established qualitative criteria, including credibility, dependability, confirmability, and transferability (Lincoln & Guba, 1985). Credibility was enhanced through prolonged engagement with the data, iterative analysis, and regular discussions among the research team to deepen interpretation. Member reflections were conducted with a subset of participants (*n* = 10), who were invited to review a written summary of the preliminary themes and provide feedback on their resonance, plausibility, and recognisability. Participants were encouraged to identify any significant omissions or aspects they felt were misrepresented in the interpretations. Their responses—shared through brief written or verbal commentary following an individual review of the summary—broadly confirmed the resonance of the emerging themes with their own experiences, while also highlighting nuances related to institutional constraints and the perceived invisibility of postural health within school structures. These insights were incorporated into the ongoing thematic development, particularly in refining the scope of Themes 1 and 3. Consistent with a reflexive thematic approach, this process was not intended to validate the findings as factually accurate, but rather to strengthen the interpretative credibility of the analysis by ensuring that the themes were meaningful and recognisable from participants' situated perspectives (Braun & Clarke, 2006, 2019). Dependability and confirmability were supported through the maintenance of an audit trail documenting analytical decisions, as well as reflexive journaling to critically examine the researchers’ assumptions and interpretative role. Transferability was addressed by providing detailed descriptions of participants, context, and research processes, enabling readers to assess the applicability of the findings to other settings. These criteria were applied in a flexible and interpretative manner, consistent with a reflexive thematic approach rather than as fixed procedural checks.

### Ethical considerations

The study was conducted in accordance with the ethical principles outlined in the Declaration of Helsinki (Medical Association, 2024). Ethical approval was obtained from the Ethics Committee of the University of Alicante (protocol: UA-2023-11-16), and all participants provided written informed consent prior to participation. Confidentiality and anonymity were ensured throughout the research process, and all identifying information was removed from transcripts and reports—referenced using anonymised codes (E01–E30).

## Results

**Figure 1.**
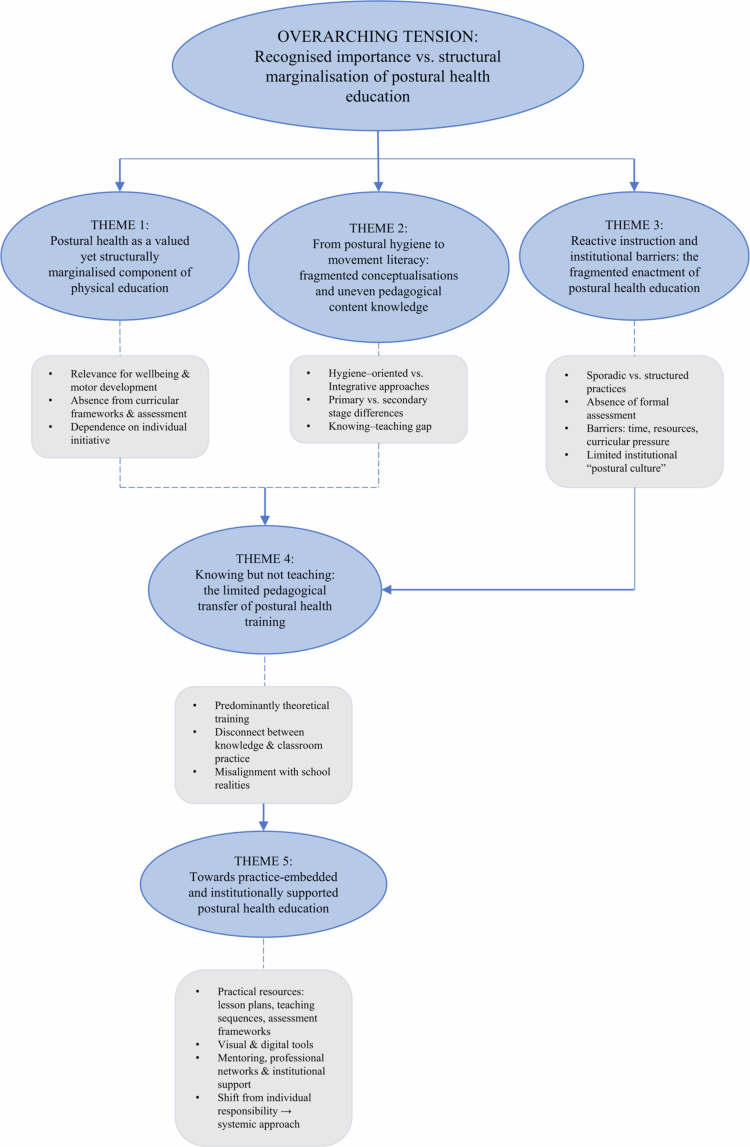
Thematic map illustrating the five interrelated themes and their relationships, generated through reflexive thematic analysis (Braun & Clarke, 2006, 2019). The overarching tension between the recognised importance and structural marginalisation of postural health education is represented at the top. Arrows indicate the interpretative relationships between themes: curricular marginalisation (Theme 1) shapes both fragmented conceptualisations (Theme 2) and inconsistent implementation (Theme 3); the knowing–teaching gap (Theme 2) is reinforced by insufficiently practice-oriented training (Theme 4); structural constraints (Theme 3) further limit the impact of training; and these converging challenges inform teachers' demand for practical, contextualised, and collaborative support (Theme 5).

The analysis generated five interrelated themes that capture how PE teachers conceptualise and enact postural health education within school contexts. These themes represent patterns of shared meaning across participants’ accounts, constructed through the dynamic interplay between those accounts and the researchers’ interpretative engagement. They also reflect variations in how teachers interpret, prioritise, and implement postural education in practice. Figure 1 provides a thematic map that visually represents the five themes and the interpretative relationships identified across participants' accounts, with the central tension between teachers' strong recognition of the importance of postural health and its marginal curricular and institutional status constituting the organising thread connecting all five themes. Themes are presented narratively below, with illustrative quotations referenced using anonymised interview codes (E01–E30).

### Theme 1: postural health as a valued yet structurally marginalised component of PE

While the marginal status of postural health in school curricula provided the initial impetus for this study, teachers' accounts revealed a more complex and nuanced picture: postural health was not merely absent or overlooked, but actively valued and yet systematically deprioritised through institutional mechanisms that teachers both identified and, in some cases, reproduced. Teachers consistently positioned postural health as a fundamental component of PE, emphasising its relevance for students’ wellbeing, motor development, and long-term physical functioning. Across interviews, postural education was framed not only as a preventive health issue but also as a foundational element influencing how students move, breathe, and engage in physical activity. As one teacher explained: “Posture affects how they move, how they breathe, and how they feel in class… If we don’t address that from a young age, it lingers in everything they do” (E08).

However, this strong recognition contrasted with its limited curricular visibility. Teachers described postural health as largely absent from formal curricular frameworks and assessment structures, resulting in a form of pedagogical marginalisation. This created a persistent tension between acknowledging its importance and being able to prioritise it within everyday teaching practice. Implementation was frequently described as dependent on individual initiative rather than institutional guidance, leading to inconsistent practices across schools. In this sense, the marginal position of postural health reflects broader institutional dynamics that shape what is considered legitimate and assessable knowledge within PE.

### Theme 2: from postural hygiene to movement literacy: fragmented conceptualisations and uneven PCK

Teachers’ conceptualisations of postural education were heterogeneous and reflected different underlying pedagogical orientations. A dominant pattern involved a hygiene-oriented perspective, in which postural education was reduced to corrective behaviours such as sitting posture or backpack use. These approaches tended to emphasise prevention through isolated recommendations rather than structured pedagogical processes: “It’s mainly about teaching them to sit properly and wear the backpack well. That’s what we focus on most” (E11).

In contrast, some teachers articulated a more integrative understanding, framing postural education as a continuum involving dynamic alignment, motor control, and body awareness. These perspectives connected postural health to broader constructs such as physical literacy and long-term movement competence: “…I understand it as global motor education: it starts when sitting, continues during movement, and consolidates with body control and core strength” (E07).

Differences between educational stages were also evident. Primary school teachers tended to emphasise basic habits and preventive messages, whereas secondary teachers more frequently referred to biomechanical or anatomical concepts. However, this increased conceptual complexity did not necessarily translate into more structured pedagogical implementation.

Across both groups, teachers’ self-assessed PCK was often described as limited or partial. Importantly, greater conceptual understanding did not consistently translate into pedagogical confidence, revealing a gap between knowing and teaching. This gap was closely linked to the nature of training experiences described by participants (Theme 4), which were often perceived as insufficiently practice-oriented.

### Theme 3: reactive instruction and institutional barriers: the fragmented enactment of postural health education

Postural health education was enacted in diverse but predominantly fragmented ways. While some teachers described structured instructional sequences focused on alignment, motor awareness, or core stability, these practices were not widespread. More commonly, postural education was integrated in an incidental manner, through sporadic corrections or brief verbal prompts during activities. This pattern reflects a shift from intentional pedagogy to reactive practice, where postural issues are addressed when they arise rather than through planned instruction. In some cases, teachers acknowledged deprioritising postural education altogether, despite recognising its importance.

A key feature of this theme was the absence of formal assessment, which contributed to the perceived marginal status of postural education. Without clear evaluation criteria, postural content remained pedagogically invisible and lacked continuity within teaching programmes: “I sometimes include postural work, like core exercises or balance drills, but it’s not something students see as part of the curriculum… I don’t assess it, so it doesn’t really count” (E14).

Teachers identified multiple interrelated barriers, including limited training, time constraints, lack of resources, and curricular pressures. These factors were not experienced in isolation but as part of a broader institutional context characterised by a limited “postural culture”, where both leadership and departmental priorities played a role in shaping what was taught. As such, implementation challenges cannot be understood solely at the individual level but must be situated within structural and organisational conditions.

### Theme 4: knowing but not teaching: the limited pedagogical transfer of postural health training

Teachers widely recognised the importance of training in postural health education, yet their experiences were often described as fragmented and predominantly theoretical. Training frequently focused on anatomical knowledge or basic biomechanical principles, with limited applicability to classroom practice: “I know how to explain what a neutral spine is or how the diaphragm works, but I wouldn’t know how to build a 4-session sequence or assess it beyond observation” (E12).

This created a disconnect between knowledge acquisition and classroom practice, reinforcing the gap between conceptual understanding and pedagogical implementation identified in Theme 2. Even teachers with additional qualifications or extensive experience expressed difficulties in translating theoretical knowledge into structured teaching strategies. Rather than functioning as a driver of pedagogical change, training was often experienced as insufficiently aligned with the realities of school contexts. This highlights the need to reconceptualise professional development not only as knowledge transmission but as a process that supports practical application, curricular integration, and sustained change in teaching practice.

### Theme 5: towards practice-embedded and institutionally supported postural health education

Teachers expressed a strong demand for practical and accessible resources that could support the integration of postural health education into everyday teaching. Across interviews, there was a clear preference for tools that bridge the gap between theory and practice, such as structured lesson plans, progressive teaching sequences, and assessment frameworks: “Resources shouldn’t just be theory. Seeing another teacher apply it with real students, I’m more likely to try it myself… That’s the kind of support we need” (E25).

Participants also highlighted the potential of visual and digital resources, including video demonstrations, movement analysis tools, and posture-related applications. These resources were valued not only for their instructional potential but also for their capacity to enhance student engagement and teacher confidence.

Beyond individual tools, teachers emphasised the importance of institutional and collaborative support. Suggestions included mentoring programmes, in-school training, and professional networks for sharing good practice across schools. These proposals reflect a shift from individual responsibility towards more systemic approaches, where the integration of postural health education is supported by organisational structures and professional communities.

## Discussion

This study explored how PE teachers conceptualise and enact postural health education within school contexts, revealing a consistent tension between its perceived importance and the limited structures supporting its implementation. While previous research has primarily focused on the design and effectiveness of postural education programmes (Anyachukwu et al., 2024; Araújo-Lima et al., 2023; Chacón-Borrego et al., 2018), this study contributes a practice-oriented perspective by examining how such content is interpreted and enacted in everyday teaching.

A central finding is the paradoxical position of postural health as both highly valued and structurally marginalised. Teachers consistently framed it as essential for students’ movement competence, wellbeing, and long-term physical development, aligning with broader conceptualisations of physical literacy (Whitehead, 2010). However, its limited presence in curricular frameworks and assessment structures constrained its implementation. This tension reflects not only curricular omissions but also broader institutional dynamics that shape what is recognised as legitimate and assessable knowledge in PE (Hardman et al., 2014; Kirk, 2009). In this sense, the marginalisation of postural health may be understood as a product of systemic prioritisation processes rather than individual teacher decisions.

Teachers’ conceptualisations of postural education further revealed a divide between hygiene-oriented approaches and more integrative, movement-based perspectives. While this duality echoes longstanding tensions between transmissive and constructivist pedagogies in PE (Aartun et al., 2022; Light, 2012), it also highlights an important gap in PCK (Ball et al., 2008; Shulman, 1987). Notably, even when teachers demonstrated conceptual understanding, they often reported difficulties in translating this knowledge into structured teaching practices. This finding suggests that pedagogical limitations cannot be reduced to knowledge deficits alone but are also shaped by the absence of practical frameworks, assessment tools, and shared pedagogical models.

The fragmented implementation patterns observed in this study—ranging from structured sessions to sporadic corrections—reinforce this interpretation. While previous research has attributed limited implementation primarily to insufficient training (Araújo-Lima et al., 2023; Chacón-Borrego et al., 2018), our findings suggest a more complex interplay of factors, including curricular pressure, limited instructional time, and uncertainty regarding effective pedagogical approaches. In this regard, the reliance on reactive, low-intensity strategies may reflect pragmatic adaptations to contextual constraints rather than a lack of teacher commitment. This interpretation invites a more cautious reading of implementation gaps, recognising that they emerge within specific institutional and organisational conditions (Domitrovich et al., 2008; Durlak & DuPre, 2008), highlighting the need to avoid reductive, single-factor explanations.

Training experiences were widely perceived as insufficiently practice-oriented, echoing evidence that isolated professional development initiatives rarely lead to sustained pedagogical change (Armour & Yelling, 2007; Garet et al., 2001; Guskey, 2002). However, it is also possible that the limited impact of training reflects a misalignment between training content and the realities of school contexts, alongside competing curricular demands that restrict opportunities for implementation. This may be compounded by potential ambiguity in the evidence base regarding effective postural interventions, which can make it difficult for teachers to identify clear, actionable strategies. In this sense, implementation challenges should not be attributed solely to deficits in teacher training, but understood as emerging from the interaction between pedagogical uncertainty, curricular pressures, and broader institutional constraints. These findings suggest that professional development should be reconceptualised as an ongoing, context-sensitive process that integrates theoretical knowledge with practical application, collaborative learning, and institutional support (Darling-Hammond et al., 2017; Vescio et al., 2008).

Teachers’ expressed need for practical resources and collaborative structures further supports this perspective. While previous studies have emphasised the role of instructional materials and digital tools (Armour & Yelling, 2007; Darling-Hammond et al., 2017; Garet et al., 2001; Guskey, 2002; Vescio et al., 2008), our findings highlight that resources alone are unlikely to produce meaningful change without supportive organisational conditions. The emphasis placed on mentoring, peer collaboration, and institutional commitment suggests that sustainable integration of postural health education requires a systemic approach that extends beyond individual teacher capacity. In this sense, the findings align with broader research on school-based health promotion, which underscores the importance of coherence between policy, curriculum, and practice (Fullan, 2016; Kirk, 2009).

Taken together, these findings suggest that improving postural health education is not solely a matter of enhancing teacher knowledge or providing additional resources, but of addressing the structural and cultural conditions that shape educational priorities. By situating teachers’ experiences within these broader contexts, this study contributes to ongoing discussions on how educational systems can either support or constrain the equitable distribution of health-related knowledge (Kickbusch, 2001; Nutbeam, 2025). Importantly, these findings should be interpreted within the specific context of the Spanish educational system, and particularly the Valencian Community. While they may resonate with broader international trends, they are not intended to be generalised uncritically across contexts. Rather, they offer context-sensitive insights that may inform future research and policy discussions in similar educational settings, taking into account the influence of local curricular structures, institutional priorities, and cultural understandings of health and PE.

## Limitations and directions for future research

This study should be interpreted in light of several limitations. First, the findings are based on self-reported data, which may be influenced by recall bias or social desirability, particularly given the professional context in which teachers may seek to present their practices in a favourable way. However, qualitative interviews remain a valuable approach for exploring participants’ reasoning, meanings, and decision-making processes. Second, the study is situated within a specific regional context—the Valencian Community in Spain—which may limit the transferability of findings to other educational systems with different curricular structures, policy environments, or cultural understandings of health and PE. Rather than aiming for generalisation, the study provides context-sensitive insights that may inform similar settings. Third, the study focuses on teachers’ perspectives and does not include direct observation of classroom practices or assessment of student outcomes. As such, the findings reflect perceived rather than enacted practices, which may differ in situ.

Future research could build on these findings by incorporating observational designs, longitudinal approaches, and multi-level analyses that examine how postural health education is implemented over time and across institutional contexts. In addition, mixed-methods studies integrating teacher perspectives with student outcomes would provide a more comprehensive understanding of the impact of postural education on learning and wellbeing. Future studies might also examine the role of digital tools and devices in supporting postural health instruction, given the strong interest expressed by teachers in technology-based resources as a means of bridging the gap between theory and practice.

## Conclusions

This study provides an in-depth qualitative account of how PE teachers conceptualise and implement postural health education within the Spanish school context, where PE is a compulsory subject across both primary and secondary educational stages. The findings reveal a consistent gap between the recognised importance of postural health and the limited curricular, pedagogical, and institutional structures supporting its implementation. Teachers highlighted the relevance of postural education for students’ movement competence and wellbeing, yet reported fragmented knowledge, inconsistent practices, and limited access to practical resources. These findings underscore the central role of PCK in translating theoretical concepts into meaningful learning experiences, while also demonstrating how teaching practices are shaped by broader institutional and curricular conditions.

By identifying interconnected intrapersonal, interpersonal, and organisational barriers, the study points to the need for more coherent and practice-oriented approaches to postural health education. In particular, the findings suggest that strengthening curricular visibility, supporting context-sensitive professional development, and fostering collaborative structures within schools may enhance the sustainability and quality of implementation. Importantly, these conclusions are grounded in a specific educational context and should be interpreted as context-sensitive insights rather than generalisable claims. Nonetheless, they contribute to broader discussions on how educational systems can better support equitable access to health-related knowledge and promote students’ long-term wellbeing, while recognising that such processes are always shaped by contextual and institutional conditions.

## Supplementary Material

Codebook.docx Codebook.docx

Code Framework.docx Code Framework.docx

## Data Availability

Due to the sensitive and identifiable nature of qualitative interview data, and because the dataset forms part of an ongoing doctoral research project, the data cannot be made publicly available. De-identified excerpts are available from the corresponding author upon reasonable request.
