# Supplementary material for: Postural health education in schools: teachers' perspectives on well-being, inequities, and institutional constraints
Source: Int J Qual Stud Health Well-being. 2026 May 14;21(1):2674331. doi: 10.1080/17482631.2026.2674331 (PMC13178034; doi:10.1080/17482631.2026.2674331)
Supplement: Codebook.docx [file ZQHW_A_2674331_SM9196.docx]

**Theme 1: Postural health as a valued yet structurally marginalised component of Physical Education**

This theme explores how Physical Education teachers position postural health as a valued yet structurally marginalised component of their practice. It captures the tension between teachers' strong recognition of postural health as educationally relevant and its limited presence in formal curricula and assessment structures. The theme reflects how personal convictions about the importance of postural education coexist with institutional mechanisms that systematically deprioritise it, offering insight into the degree of curricular legitimisation attributed to postural health content.

1. ***Perceived importance*.** This code captures teachers’ assessments of the relevance of postural education in pupils’ overall development. It focuses on the degree of importance they attribute to it as an educational component within the subject of Physical Education.
   1. *High importance*. The teacher regards postural education as a fundamental component in the holistic development of students, emphasising its impact on health, wellbeing, and injury prevention.
   2. *Moderate importance*. The usefulness of postural education is acknowledged, yet it is not afforded a central role. It may be viewed as a complementary or secondary element within the teaching programme.
   3. *Low importance*. The teacher downplays the relevance of postural education, considering it to be of limited significance or dispensable within the school context.
2. ***Curricular integration of postural content*.** This code examines how teachers perceive the presence of postural education within the official curriculum and in their own pedagogical practices.
   1. *Curricular inclusion*. Postural content is explicitly integrated into teaching plans or curricular documents. The teacher addresses it in a systematic and planned manner.
   2. *Curricular insufficiency.* A lack or weakness is identified in the curricular inclusion of postural education. The teacher notes that it is insufficiently represented in official documents or that its implementation is sporadic and lacks structure*.*

**Theme 2: From postural hygiene to movement literacy: fragmented conceptualisations and uneven pedagogical content knowledge**

This theme explores the diversity of conceptual frameworks through which Physical Education teachers understand postural education, ranging from hygiene-oriented to integrative, movement-based perspectives. It also examines teachers' self-assessed pedagogical content knowledge and the gap between conceptual understanding and pedagogical confidence. The theme reveals how different epistemological orientations underpin teachers' professional reasoning and shape their capacity to translate knowledge into meaningful instructional practice.

1. ***Conceptual understandings*.** This code captures the underlying conceptual frameworks through which teachers interpret the nature and purpose of postural education.
   1. *Hygiene-oriented*. Postural education is understood primarily in terms of bodily hygiene, ergonomics, and the prevention of musculoskeletal disorders.
   2. *Integrative*. Postural education is viewed as a multidimensional construct linked to movement literacy, motor control, body awareness, and broader physical literacy. It integrates psychosocial wellbeing and long-term movement competence, moving beyond hygiene-oriented prevention.
2. ***Perceived pedagogical knowledge*.** This code reflects teachers’ self-assessed levels of pedagogical knowledge and confidence in teaching postural education effectively.
   1. *Insufficient*. The teacher feels inadequately prepared or lacks the necessary knowledge to teach postural education.
   2. *Sufficient*. The teacher considers their pedagogical knowledge to be adequate for delivering basic postural education.
   3. *Advanced*. The teacher demonstrates a high level of expertise and confidence in teaching postural education, often supported by specialised training or experience.
   4. *Partial*. The teacher possesses some relevant knowledge but acknowledges gaps or limitations in their pedagogical approach.

**Theme 3: Reactive instruction and institutional barriers: the fragmented enactment of postural health education**

This theme examines how Physical Education teachers enact postural health education in practice, capturing a pattern characterised predominantly by reactive instruction and fragmented implementation. While some teachers describe structured approaches, the dominant mode involves incidental corrections and informal integration. The theme also identifies the interrelated institutional and contextual barriers—including limited training, time constraints, resource scarcity, and absence of a postural culture—that constrain intentional pedagogical action.

1. ***Classroom implementation*.** This code refers to the ways in which postural education is enacted within Physical Education lessons, including the degree of intentionality, integration, and assessment.
   1. *Planned and explicit*. Postural education is delivered through structured activities, with clear objectives and intentional planning.
   2. *Transversal integration*. Postural content is embedded across other units or topics, without being the central focus.
   3. *Not implemented*. The teacher does not include postural education in their teaching practice.
   4. *Informal implementation*. Postural education is addressed sporadically or reactively, such as through spontaneous corrections or brief mentions.
   5. *Deprioritised content*. The teacher acknowledges the value of postural education but does not prioritise it due to competing curricular demands.
   6. *Not formally assessed*. Postural education is taught but not evaluated through formal assessment tools or criteria.
2. ***Barriers*.** This code captures the perceived challenges and limitations that prevent teachers from effectively implementing postural education in their teaching practice.
   1. *Lack of specific training*. Teachers report insufficient professional preparation or specialised training in postural education.
   2. *Lack of institutional priority*. Postural education is not prioritised by the school or educational authorities, limiting its visibility and support.
   3. *Lack of material resources*. Teachers cite the absence of appropriate equipment, materials, or teaching aids to support postural education.
   4. *Time constraints.* Limited instructional time prevents the inclusion of postural education within the curriculum.
   5. *Lack of postural culture in the school.* The broader school environment does not promote or reinforce postural awareness, making implementation more difficult.

**Theme 4: Knowing but not teaching: the limited pedagogical transfer of postural health training**

This theme explores teachers' training experiences in postural education and examines why theoretical knowledge does not translate into structured pedagogical practice. It captures the theory-practice disconnect that characterises many teachers' professional trajectories: while training has provided conceptual or anatomical knowledge, it has rarely equipped teachers with the practical frameworks, lesson structures, or assessment tools needed for sustained classroom implementation. The theme highlights the need to reconceptualise professional development as practice-oriented and contextually embedded.

1. ***Training experience*.** This code refers to the types of training that Physical Education teachers have received in relation to postural education, including formal qualifications, ad hoc learning, and self-directed efforts.
   1. *Formal initial teacher education*. Postural education was included as part of the teacher’s university-level or accredited initial training programme.
   2. *Occasional online training*. Teachers have participated in sporadic online courses or webinars related to postural education, often without follow-up or certification.
   3. *Continuing professional development*. Postural education has been addressed through structured continuing professional development activities, such as workshops, seminars, or in-service training.
   4. *Informal or self-guided training*. Teachers have independently sought information or resources on postural education, outside of formal training contexts.
2. ***Pedagogical impact*.** This code captures teachers’ perceptions of how their training has influenced their ability to teach postural education effectively.
   1. *Perceived as sufficient*. The teacher feels that their training has adequately prepared them to deliver postural education in the classroom.
   2. *Perceived as insufficient*. The teacher believes their training was lacking or inadequate for the demands of teaching postural education.
   3. *Theory-practice disconnect*. The teacher identifies a gap between theoretical knowledge acquired during training and the practical realities of classroom implementation.

**Theme 5: Towards practice-embedded and institutionally supported postural health education**

This theme captures teachers’ reflections on what is required to enhance the implementation of postural education in schools. It includes both perceived needs —such as training and institutional support— and preferred types of resources that would facilitate more effective teaching. The theme reveals a desire for practical, accessible, and context-sensitive support mechanisms.

1. ***Perceived needs*.** This code refers to the specific areas where teachers feel support is lacking and which they consider essential for improving the delivery of postural education.
   1. *Practical training*. Teachers express the need for hands-on, applicable training that equips them with concrete strategies for teaching postural education.
   2. *Institutional awareness*. Teachers highlight the importance of broader institutional recognition and prioritisation of postural education within the educational system.
   3. *Practice-sharing network*. Teachers value opportunities to exchange experiences, strategies, and resources with peers through collaborative platforms or communities of practice.
2. ***Preferred resources*.** This code identifies the types of resources that teachers consider most useful or desirable for supporting postural education in their teaching practice.
   1. *Simple teaching materials*. Teachers prefer straightforward, easy-to-use materials that can be readily integrated into existing lessons.
   2. *Comprehensive teaching materials*. Teachers request more in-depth and structured resources that offer full lesson plans, theoretical background, and assessment tools*.*
   3. *No extra materials*. Teachers who express confidence in their current approach and report no perceived need for additional resources.
   4. *Mentoring or expert support*. Teachers express interest in receiving guidance from specialists or experienced colleagues to improve their practice.
   5. *Digital tools*. Teachers suggest that digital platforms, apps, video demonstrations, and movement analysis tools could enhance both the accessibility and engagement of postural health instruction, and could help bridge the gap between theoretical knowledge and practical application.
