# Supplementary material for: Postural health education in schools: teachers' perspectives on well-being, inequities, and institutional constraints
Source: Int J Qual Stud Health Well-being. 2026 May 14;21(1):2674331. doi: 10.1080/17482631.2026.2674331 (PMC13178034; doi:10.1080/17482631.2026.2674331)
Supplement: Code Framework.docx [file ZQHW_A_2674331_SM9195.docx]

| **Project Title** | **Core Inquiry Area** | **Research Questions** | **Theme** | **Codes** | **Subcodes** |
| --- | --- | --- | --- | --- | --- |
| School-Based Postural Health Education: Physical Education Teachers’ Beliefs, Practices, and Professional Development Needs | How do Physical Education teachers perceive the importance and curricular relevance of postural education? | As a Physical Education teacher, how do you perceive the importance of postural education in student development?  Do you think postural education holds adequate weight within the Physical Education curriculum? Would you give it more or less relevance? Why? | Postural health as a valued yet structurally marginalised component of Physical Education | 1. Perceived importance | 1.1. High importance  1.2. Moderate importance  1.3. Low importance |
|  |  |  |  | 2. Curricular integration of postural content | 2.1. Curricular inclusion  2.2. Curricular insufficiency |
|  | What are Physical Education teachers’ conceptual understandings and self-assessed knowledge of postural education? | How would you define postural education? What do you consider its main objectives to be?  How would you describe your level of knowledge about postural education? In which areas do you feel more confident, and where do you think you need further support or training? | From postural hygiene to movement literacy: fragmented conceptualisations and uneven pedagogical content knowledge | 3. Conceptual understandings | 3.1. Hygiene-oriented  3.2. Integrative |
|  |  |  |  | 4. Perceived pedagogical knowledge | 4.1. Insufficient  4.2. Sufficient  4.3. Advanced  4.4. Partial |
|  | How is postural education actually implemented in Physical Education classes, and what barriers affect its practice? | How do you incorporate postural education into your Physical Education classes? What strategies or activities do you use to address this topic with your students?  What barriers have you encountered that make it difficult to implement postural education in your teaching (*e. g.*, time constraints, lack of training, limited resources, curricular priorities)? | Reactive instruction and institutional barriers: the fragmented enactment of postural health education | 5. Classroom implementation | 5.1. Planned and explicit  5.2. Transversal integration  5.3. Not implemented  5.4. Informal implementation  5.5. Deprioritised content  5.6. Not formally assessed |
|  |  |  |  | 6. Barriers | 6.1. Lack of specific training  6.2. Lack of institutional priority  6.3. Lack of material resources  6.4. Time constraints  6.5. Lack of postural culture in the school |

| **Project Title** | **Core Inquiry Area** | **Research Questions** | **Theme** | **Codes** | **Subcodes** |
| --- | --- | --- | --- | --- | --- |
| School-Based Postural Health Education: Physical Education Teachers’ Beliefs, Practices, and Professional Development Needs | What training have Physical Education teachers received in postural education, and how do they evaluate its adequacy? | What kind of specific training have you received on postural education? Do you feel it has been sufficient for your teaching practice?  Do you consider your current training adequate to address postural education effectively in the classroom? Why or why not? | Knowing but not teaching: the limited pedagogical transfer of postural health training | 7. Training experience | 7.1. Formal initial teacher education  7.2. Occasional online training  7.3. Continuing professional development  7.4. Informal or self-guided training |
|  |  |  |  | 8. Pedagogical impact | 8.1. Perceived as sufficient  8.2. Perceived as insufficient  8.3. Theory-practice disconnect |
|  | What professional development needs and resource preferences do Physical Education teachers express regarding postural education? | Based on your experience, what changes or improvements would you suggest to make postural education more effectively implemented in schools?  Would you be interested in receiving specific teaching resources on postural education? What types of materials do you think would be most useful (*e. g.*, guides, videos, practical sessions, online training)? | Towards practice-embedded and institutionally supported postural health education | 9. Perceived needs | 9.1. Practical training  9.2. Institutional awareness  9.3. Practice-sharing network |
|  |  |  |  | 10. Preferred resources | 10.1. Simple teaching materials  10.2. Comprehensive teaching materials  10.3. No extra materials  10.4. Mentoring or expert support  10.5. Digital tools |
